# Supplementary material for: Machine Learning Accelerates Crystallization for Structure Determination
Source: Angew Chem Int Ed Engl. 2026 May 4;65(25):e1218503. doi: 10.1002/anie.1218503 (PMC13266958; doi:10.1002/anie.1218503)

checkCIF/PLATON report

Structure factors have been supplied for datablock(s) lcz112

THIS REPORT IS FOR GUIDANCE ONLY. IF USED AS PART OF A REVIEW PROCEDURE FOR PUBLICATION, IT SHOULD NOT REPLACE THE EXPERTISE OF AN EXPERIENCED CRYSTALLOGRAPHIC REFEREE.

No syntax errors found. CIF dictionary Interpreting this report

Datablock: lcz112

Bond precision:	C-C = 0.0208 A		Wavelength=1.54184
Cell:	a=33.0301(5)	b=12.9921(2)	c=23.7486(4)
	alpha=90	beta=104.652(2)	gamma=90
Temperature:	100 K		

	Calculated	Reported
Volume	9859.8(3)	9859.8(3)
Space group	C 2	C 1 2 1
Hall group	C 2y	C 2y
Moiety formula	C131 H159 Ag9 F54 N20 O32, C15 H3 Ag3 F18 N6	4(C7.5 H1.5 Ag1.5 F9 N3), C43 H75 N O16
Sum formula	C146 H162 Ag12 F72 N26 O32	C73 H81 Ag6 F36 N13 O16
Mr	5455.47	2727.72
Dx,g cm-3	1.838	1.838
Z	2	4
Mu (mm-1)	10.583	10.583
F000	5376.0	5376.0
F000’	5400.21	
h,k,lmax	41,16,30	41,15,30
Nref	21188[11073]	17713
Tmin,Tmax	0.207,0.204	0.791,1.000
Tmin’	0.132	

Correction method= # Reported T Limits: Tmin=0.791 Tmax=1.000
AbsCorr = MULTI-SCAN

```
Data completeness= 1.60/0.84        Theta(max)= 78.328

                                                          wR2(reflections)=
R(reflections)= 0.0548( 15096)                            0.1382( 17713)

S = 1.075                     Npar= 1317
```

The following ALERTS were generated. Each ALERT has the format
test-name_ALERT_alert-type_alert-level.
Click on the hyperlinks for more details of the test.

Alert level B

```
PLAT342_ALERT_3_B Low Bond Precision on  C-C Bonds ..............    0.02076 Ang.
```

Author Response: Disordered structure.

Alert level C

```
PLAT041_ALERT_1_C Calc. and Reported SumFormula    Strings  Differ     Please Check
              Calc: C146 H162 Ag12 F72 N26 O32
              Rep.: C73 H81 Ag6 F36 N13 O16
PLAT042_ALERT_1_C Calc. and Reported MoietyFormula Strings  Differ     Please Check
              Calc: C131 H159 Ag9 F54 N20 O32, C15 H3 Ag3 F18 N6
              Rep.: 4(C7.5 H1.5 Ag1.5 F9 N3), C43 H75 N O16
PLAT213_ALERT_2_C Atom F02K            has ADP max/min Ratio .....        3.4 prolat
PLAT213_ALERT_2_C Atom F03Z            has ADP max/min Ratio .....        3.5 prolat
PLAT220_ALERT_2_C NonSolvent   Resd 1  C   Ueq(max)/Ueq(min) Range        4.1 Ratio
PLAT220_ALERT_2_C NonSolvent   Resd 1  F   Ueq(max)/Ueq(min) Range        3.8 Ratio
PLAT234_ALERT_4_C Large Hirshfeld Difference F00X     --C03F     .       0.16 Ang.
PLAT234_ALERT_4_C Large Hirshfeld Difference F01T     --C03W     .       0.25 Ang.
PLAT234_ALERT_4_C Large Hirshfeld Difference F02O     --C03I     .       0.18 Ang.
PLAT234_ALERT_4_C Large Hirshfeld Difference F043     --C2       .       0.24 Ang.
PLAT234_ALERT_4_C Large Hirshfeld Difference N02A     --C03B     .       0.18 Ang.
PLAT234_ALERT_4_C Large Hirshfeld Difference C02V     --C03O     .       0.22 Ang.
PLAT234_ALERT_4_C Large Hirshfeld Difference C03D     --C03U     .       0.22 Ang.
PLAT241_ALERT_2_C High   'MainMol' Ueq as Compared to Neighbors of       C041 Check
PLAT250_ALERT_2_C Large U3/U1 Ratio for <U(i,j)> Tensor(Resd    2)        2.1 Note
PLAT360_ALERT_2_C Short  C(sp3)-C(sp3) Bond  C03S     - C041     .       1.41 Ang.
PLAT413_ALERT_2_C Short Inter XH3 .. XHn     H03Z     ..H03S     .       2.14 Ang.
                                          1/2-x,1/2+y,1-z  =      4_556 Check
PLAT601_ALERT_2_C Unit-Cell Contains Solvent Accessible VOIDS .LE.         43 Ang**3
PLAT911_ALERT_3_C Missing FCF Refl Between Thmin & STh/L=    0.600         37 Report
                0  8  0,   2  8  0,   4  0  0,  37  3  0,  -9  7  1,  -4  8  1,
                1  1  1,  37  3  1,  -9  7  2,  -2  0  2,  -2  8  2, -13  7  3,
               -9  7  3,  -6  6  3, -30 10  4,  -7  7  5,   1  7  5,  -5  7  6,
               -3  7  6,  -1  7  6,   5 13 12,  30  0 12, -24  2 14,  -1 13 15,
                1 13 15,  17  9 16,  25  1 16,  26  0 16, -23  9 18, -23  9 19,
                   (    7 More Missing: see the .ckf listing file)
PLAT915_ALERT_3_C No Flack x Check Done: Low Friedel Pair Coverage         70 %
PLAT971_ALERT_2_C Check Calcd Resid. Dens.  0.88Ang From Ag07            2.20 eA-3
PLAT972_ALERT_2_C Check Calcd Resid. Dens.  0.79Ang From Ag07           -1.69 eA-3
```

Alert level G

```
PLAT002_ALERT_2_G Number of Distance or Angle Restraints on AtSite          4 Note
PLAT003_ALERT_2_G Number of Uiso or U(i,j)  Restrained non-H-Atoms         14 Report
PLAT007_ALERT_5_G Number of Unrefined Donor-H Atoms .............           4 Report
              H00N  H00S  H00Z  H01D
PLAT045_ALERT_1_G Calculated and Reported Z Differ by a Factor ...      0.500 Check
PLAT083_ALERT_2_G SHELXL Second Parameter in WGHT  Unusually Large      28.40 Why ?
PLAT172_ALERT_4_G The CIF-Embedded .res File Contains DFIX Records          3 Report
PLAT177_ALERT_4_G The CIF-Embedded .res File Contains DELU Records          3 Report
PLAT178_ALERT_4_G The CIF-Embedded .res File Contains SIMU Records          2 Report
PLAT186_ALERT_4_G The CIF-Embedded .res File Contains ISOR Records          2 Report
PLAT242_ALERT_2_G Low    'MainMol' Ueq as Compared to Neighbors of         C2 Check
PLAT242_ALERT_2_G Low    'MainMol' Ueq as Compared to Neighbors of       C02E Check
PLAT242_ALERT_2_G Low    'MainMol' Ueq as Compared to Neighbors of       C02Y Check
PLAT242_ALERT_2_G Low    'MainMol' Ueq as Compared to Neighbors of       C03F Check
PLAT242_ALERT_2_G Low    'MainMol' Ueq as Compared to Neighbors of       C03I Check
PLAT242_ALERT_2_G Low    'MainMol' Ueq as Compared to Neighbors of       C03T Check
PLAT242_ALERT_2_G Low    'MainMol' Ueq as Compared to Neighbors of       C03U Check
PLAT242_ALERT_2_G Low    'MainMol' Ueq as Compared to Neighbors of       C03X Check
PLAT242_ALERT_2_G Low    'MainMol' Ueq as Compared to Neighbors of       C01F Check
PLAT242_ALERT_2_G Low    'MainMol' Ueq as Compared to Neighbors of       C02G Check
PLAT242_ALERT_2_G Low    'MainMol' Ueq as Compared to Neighbors of       C02X Check
PLAT432_ALERT_2_G Short Inter X...Y Contact  O019     ..C032     .       2.95 Ang.
                                                   1-x,y,-z  =      2_655 Check
PLAT432_ALERT_2_G Short Inter X...Y Contact  O019     ..C035     .       2.97 Ang.
                                                      x,y,z  =      1_555 Check
PLAT720_ALERT_4_G Number of Unusual/Non-Standard Labels ..........        224 Note
              Ag01    Ag02    Ag03    Ag04    Ag05    Ag06    Ag07    O008
              O009    O00A    O00B    O00C    F00D    F00E    O00F    N00G
              F00H    F00I    N00J    F00K    O00L    O00M    O00N    H00N
              F00O    N00P    F00Q    F00R    O00S    H00S    F00T    N00U
              F00V    F00W    F00X    O00Y    O00Z    H00Z    F010    N011
              F012    F013    N014    N015    F017    O018    O019    C01A
              H01A    N01B    N01C    O01D    H01D    F01E    C01F    F01G
              C01H    N01I    F01J    N01K    C01L    F01M    C01N    H01B
              H01C    H01E    C01O    H01O    O01P    C01Q    C01R    H01F
              H01G    C01S    H01S    F01T    C01U    H01U    C01V    H01H
              H01I    H01J    N01W    C01X    H01X    F01Y    C01Z    C020
              H020    F021    C022    H02A    H02B    F023    C024    C025
              H02C    H02D    C026    C027    H02E    H02F    C028    H028
              C029    H029    N02A    F02B    C02C    H02     C02D    C02E
              F02F    C02G    C02H    H02H    C02I    H02Y    C02J    H02G
              H02I    H02J    F02K    C02L    H02K    H02L    H02M    F02M
              C02N    H02N    F02O    C02P    H02O    H02P    C02Q    H02Q
              H02R    H02S    C02R    C02S    H02Z    C02T    C02U    H02T
              H02U    H02V    C02V    C02W    H02W    H02X    C02X    C02Y
              C02Z    C030    C031    C032    C033    C034    C035    H035
              C036    H03A    H03B    H03C    C037    H037    C038    H038
              C039    H039    C03A    C03B    H03D    H03E    H03F    C03C
              H03G    H03H    H03I    C03D    C03E    H03     C03F    F03G
              C03H    H03J    H03K    H03L    C03I    C03J    H03M    H03N
              H03O    C03K    C03L    H03P    F03M    F03N    C03O    H03Z
              F03P    F03Q    C03R    H03R    C03S    H03Q    H03S    H03T
              C03T    C03U    C03V    H03U    H03V    H03W    C03W    C03X
              C03Y    H03X    H03Y    F03Z    C041    H04A    H04B    F043
PLAT779_ALERT_4_G Suspect or Irrelevant (Bond) Angle(s) in CIF ...       0.00 Deg.
              AG04 -N01B -AG04    2_656   1_555   1_555 ......     #   53 Check
```

```
PLAT791_ALERT_4_G Model has Chirality at C01A       (Sohncke SpGr)          R Verify
PLAT791_ALERT_4_G Model has Chirality at C01O       (Sohncke SpGr)          R Verify
PLAT791_ALERT_4_G Model has Chirality at C01S       (Sohncke SpGr)          R Verify
PLAT791_ALERT_4_G Model has Chirality at C01U       (Sohncke SpGr)          R Verify
PLAT791_ALERT_4_G Model has Chirality at C01X       (Sohncke SpGr)          S Verify
PLAT791_ALERT_4_G Model has Chirality at C020       (Sohncke SpGr)          S Verify
PLAT791_ALERT_4_G Model has Chirality at C026       (Sohncke SpGr)          R Verify
PLAT791_ALERT_4_G Model has Chirality at C028       (Sohncke SpGr)          S Verify
PLAT791_ALERT_4_G Model has Chirality at C029       (Sohncke SpGr)          S Verify
PLAT791_ALERT_4_G Model has Chirality at C02H       (Sohncke SpGr)          R Verify
PLAT791_ALERT_4_G Model has Chirality at C02N       (Sohncke SpGr)          S Verify
PLAT791_ALERT_4_G Model has Chirality at C030       (Sohncke SpGr)          S Verify
PLAT791_ALERT_4_G Model has Chirality at C033       (Sohncke SpGr)          R Verify
PLAT791_ALERT_4_G Model has Chirality at C037       (Sohncke SpGr)          R Verify
PLAT791_ALERT_4_G Model has Chirality at C038       (Sohncke SpGr)          R Verify
PLAT791_ALERT_4_G Model has Chirality at C039       (Sohncke SpGr)          R Verify
PLAT791_ALERT_4_G Model has Chirality at C03L       (Sohncke SpGr)          R Verify
PLAT791_ALERT_4_G Model has Chirality at C03R       (Sohncke SpGr)          R Verify
PLAT860_ALERT_3_G Number of Least-Squares Restraints ............          86 Note
PLAT910_ALERT_3_G Missing FCF Reflection(s) Below Theta(Min)[Deg]=       2.77 Note
                0  0  1,
PLAT912_ALERT_4_G Missing # of FCF Reflections Above STh/L=  0.600        338 Note
PLAT913_ALERT_3_G Missing # of Very Strong Reflections in FCF ....          1 Note
                4  0  0,
PLAT933_ALERT_2_G Number of HKL-OMIT Records in Embedded .res File          7 Note
              -18  2  9, -18  2 10,   1  3  4,   1  7  5,   4  6  3,  11-15  1,
               26 -4 15,
PLAT941_ALERT_3_G Average HKL Measurement Multiplicity ..........         3.2 Low
PLAT969_ALERT_5_G The 'Henn et al.' R-Factor-gap value ..........       2.023 Note
              Predicted wR2: Based on SigI**2  6.83 or SHELX Weight 12.86
PLAT978_ALERT_2_G Number C-C Bonds with Positive Residual Density.          0 Info
```

```
   0 ALERT level A = Most likely a serious problem - resolve or explain
   1 ALERT level B = A potentially serious problem, consider carefully
  22 ALERT level C = Check. Ensure it is not caused by an omission or oversight
  50 ALERT level G = General information/check it is not something unexpected

   3 ALERT type 1 CIF construction/syntax error, inconsistent or missing data
  29 ALERT type 2 Indicator that the structure model may be wrong or deficient
   7 ALERT type 3 Indicator that the structure quality may be low
  32 ALERT type 4 Improvement, methodology, query or suggestion
   2 ALERT type 5 Informative message, check
```

Validation response form

Please find below a validation response form (VRF) that can be filled in and pasted into your CIF.

```
# start Validation Reply Form
_vrf_PLAT041_lcz112
;
PROBLEM: Calc. and Reported SumFormula    Strings  Differ     Please Check
RESPONSE: ...
;
_vrf_PLAT042_lcz112
;
```

```
PROBLEM: Calc. and Reported MoietyFormula Strings  Differ     Please Check
RESPONSE: ...
;
_vrf_PLAT213_lcz112
;
PROBLEM: Atom F02K            has ADP max/min Ratio .....        3.4 prolat
RESPONSE: ...
;
_vrf_PLAT220_lcz112
;
PROBLEM: NonSolvent   Resd 1  C   Ueq(max)/Ueq(min) Range        4.1 Ratio
RESPONSE: ...
;
_vrf_PLAT234_lcz112
;
PROBLEM: Large Hirshfeld Difference F00X     --C03F     .       0.16 Ang.
RESPONSE: ...
;
_vrf_PLAT241_lcz112
;
PROBLEM: High   'MainMol' Ueq as Compared to Neighbors of       C041 Check
RESPONSE: ...
;
_vrf_PLAT250_lcz112
;
PROBLEM: Large U3/U1 Ratio for <U(i,j)> Tensor(Resd    2)        2.1 Note
RESPONSE: ...
;
_vrf_PLAT360_lcz112
;
PROBLEM: Short  C(sp3)-C(sp3) Bond  C03S     - C041     .       1.41 Ang.
RESPONSE: ...
;
_vrf_PLAT413_lcz112
;
PROBLEM: Short Inter XH3 .. XHn     H03Z     ..H03S     .       2.14 Ang.
RESPONSE: ...
;
_vrf_PLAT601_lcz112
;
PROBLEM: Unit-Cell Contains Solvent Accessible VOIDS .LE.         43 Ang**3
RESPONSE: ...
;
_vrf_PLAT911_lcz112
;
PROBLEM: Missing FCF Refl Between Thmin & STh/L=    0.600         37 Report
RESPONSE: ...
;
_vrf_PLAT915_lcz112
;
PROBLEM: No Flack x Check Done: Low Friedel Pair Coverage         70 %
RESPONSE: ...
;
_vrf_PLAT971_lcz112
;
PROBLEM: Check Calcd Resid. Dens.  0.88Ang From Ag07            2.20 eA-3
RESPONSE: ...
```

```
;
_vrf_PLAT972_lcz112
;
PROBLEM: Check Calcd Resid. Dens.  0.79Ang From Ag07           -1.69 eA-3
RESPONSE: ...
;
# end Validation Reply Form
```

It is advisable to attempt to resolve as many as possible of the alerts in all categories. Often the minor alerts point to easily fixed oversights, errors and omissions in your CIF or refinement strategy, so attention to these fine details can be worthwhile. It is up to the individual to critically assess their own results and, if necessary, seek expert advice.

PLATON version of 17/09/2025; check.def file version of 17/09/2025

duplicate check

No duplication found

Datablock lcz112 - ellipsoid plot

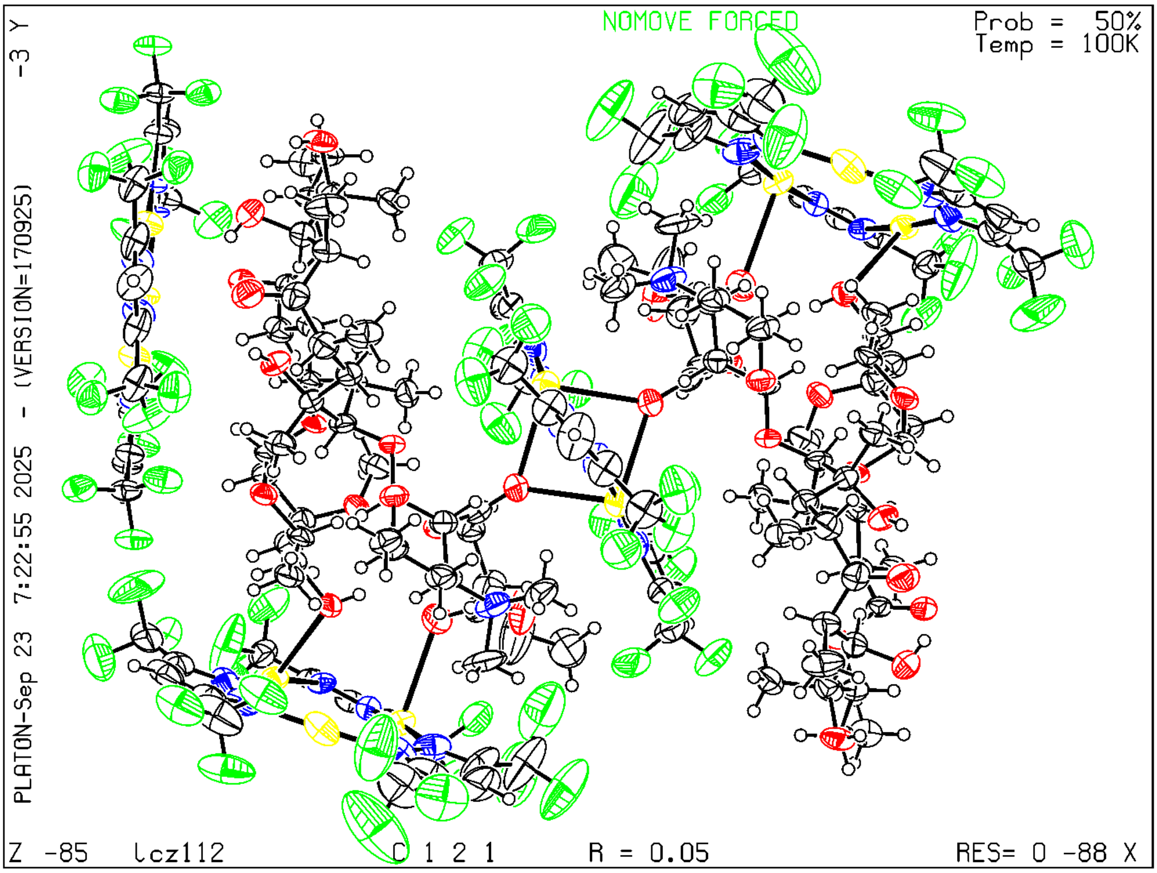

Supplement: Supplementary file 2 — Supporting File 2: anie72462‐sup‐0002‐DataFile.zip. [file ANIE-65-e1218503-s002.zip › supplementary_materials_crystallographic_data submit.zip!/cif_and_checkcif(1-114)/Ag3Pz3_112/Ag3Pz3_112_checkcif.pdf]
